# Use of Er: YAG laser and self-adhesive composite for decayed deciduous teeth treatment

**DOI:** 10.6026/973206300213084

**Published:** 2025-09-30

**Authors:** Harinder Singh Mangat, Rajkumari Sarada Devi, Meetu Mathur, Saima Ali, Ratna Yumkham, Satyabrata Das, Miral Mehta, Dhirendra Kumar Singh

**Affiliations:** 1 Department of Dental Surgery, General Dentist (India)/Registered Dental Assistant level 2, 506-280, Williamstown Close NW, Airdrie, Alberta, Canada; 2 Department of Paediatric and Preventive Dentistry, Dental College, Regional Institute of Medical Sciences, Imphal, Manipur, India; 3 Department of Conservative Dentistry and Endodontics, Rajasthan Dental College and Hospital, Nirwan University, Jaipur, Rajasthan, India; 4 Department of Public Health Dentistry, Maharishi Markandeshwar Deemed University, Mullana, Ambala, Haryana, India; 5 Dental Surgeon, CHC Dasarathpur, Jajpur, Odisha, India; 6 Department of Pediatric and Preventive Dentistry, Karnavati School of Dentistry, Karnavati University, Gandhinagar, Gujarat, India; 7 Department of Periodontology, Kalinga Institute of Dental Sciences, KIIT Deemed to be University, Bhubaneswar, Odisha, India

**Keywords:** Er: YAG laser, primary molars, caries removal, self-adhesive composite, pediatric dentistry

## Abstract

The Er: YAG laser cavity preparation with self-adhesive flowable composite to conventional rotary preparation with adhesive protocols in 120 primary molars is of interest. Group A showed longer preparation time but better patient cooperation and less anesthetic use. Restoration outcomes at 12 months were comparable in retention, marginal adaptation and secondary caries. Thus, Er: YAG laser approach offers a child-friendly alternative with clinical efficacy.

## Background:

Dental caries remains the most prevalent chronic disease affecting children worldwide, with a substantial global burden that compromises oral health, general well-being and quality of life. Left untreated, carious lesions in primary teeth can lead to pain, pulpitis, abscess formation and early tooth loss, potentially disrupting mastication, phonetics and the maintenance of arch integrity during the mixed dentition phase [1]. The conventional management of dental caries in children often involves the use of high-speed rotary instruments for cavity preparation. However, these mechanical techniques generate vibration, noise and thermal stimuli that can evoke fear and discomfort in pediatric patients, sometimes necessitating behavioral interventions or local anesthesia to complete treatment [2]. In pursuit of more child-friendly alternatives, laser-assisted caries removal-particularly with Erbium-doped Yttrium Aluminum Garnet (Er: YAG) lasers-has gained popularity. The Er: YAG laser emits a 2940 nm wavelength that is highly absorbed by water and hydroxyapatite, facilitating effective ablation of hard dental tissues with minimal collateral thermal damage [3]. Its non-contact mechanism and absence of drilling noise contribute to enhanced patient comfort and cooperation, making it well-suited for pediatric dentistry [4]. Studies have demonstrated that Er: YAG laser cavity preparation yields smear layer-free surfaces, promoting favorable adhesive conditions and maintaining pulpal health in primary teeth [5]. Simultaneously, restorative materials have evolved to meet the demands of simplified, efficient application in uncooperative children. Self-adhesive flowable composites, such as those incorporating acidic functional monomers, eliminate the need for separate etching and bonding steps, thereby reducing clinical time and technique sensitivity [6]. These materials exhibit promising bond strengths to enamel and dentin and have shown satisfactory performance in Class I restorations under conventional preparation protocols [7]. However, the existing body of evidence evaluating self-adhesive composites primarily involves cavities prepared with rotary instruments, leaving their compatibility with laser-prepared surfaces underexplored. Previous in vitro studies assessing Er: YAG laser efficacy has largely focused on ablation rates, surface topography and micromorphology of enamel and dentin [8], with limited clinical translation regarding restoration longevity and patient-centered outcomes. Moreover, although short-term clinical trials have validated the acceptability and marginal integrity of self-adhesive flowable composites in pediatric restorations, no randomized controlled trials have investigated their combined use with Er: YAG laser-prepared cavities in the primary dentition [9]. Therefore, it is of interest to address this gap by comparing the clinical performance and patient-centered outcomes of Er: YAG laser cavity preparation followed by self-adhesive flowable composite placement, against the conventional rotary bur and etch-and-rinse adhesive technique in restoring occlusal caries in primary molars.

## Materials and Methods:

## Study design:

Prospective, single-blind, randomized controlled trial.

## Sample size and selection:

60 healthy children (5-8 years) presenting at the Pediatric Dentistry Clinic with bilateral occlusal caries in primary molars; 120 teeth allocated equally to two groups via computer-generated randomization.

## Inclusion criteria:

Frankl behavior rating ≥ 3, carious lesions limited to enamel and outer dentin (ICDAS 3-4), absence of pulp involvement on radiographs.

## Exclusion criteria:

Systemic illness, uncooperative behavior, prior laser treatment.

## Equipment and materials:

[1] Er: YAG laser (LiteTouch, Syneron, Israel): enamel (300 mJ/pulse, 20 Hz); dentin (200 mJ/pulse, 20 Hz); sapphire tip Ø 1.3 mm; air/water spray ratio 8:1.

[2] Self-adhesive flowable composite (Vertise^TM^ Flow, Kerr, USA).

[3] High-speed dental turbine with carbide bur (ISO 806 023; Dentsply, USA).

[4] Etch-and-rinse adhesive system (Single Bond^TM^, 3M ESPE, USA) and conventional flowable composite (Filtek^TM^ Z250 Flow, 3M ESPE).

## Experimental procedures:

Group A: Laser ablation under rubber dam; cavity margins refined with laser tip; self-adhesive composite applied per manufacturer (2 mm increments, 20 s light cure).

Group B: Conventional bur preparation; 37% phosphoric acid etch (15 s), rinse, air-dry; adhesive application (Single Bond^TM^, 20 s, light curing), composite placement and curing identical to Group A.

## Statistical methods:

Preparation time and restoration time were recorded with a stopwatch. Patient cooperation was assessed by an independent observer using Frankl's scale. Anesthetic use logged. Restorations evaluated at 6 and 12 months using modified USPHS criteria: retention, marginal adaptation, marginal discoloration, secondary caries and postoperative sensitivity. Categorical variables compared by chi-square test; continuous variables by independent t-test; significance set at p < 0.05.

## Results:

All 120 restorations completed; 6 teeth (5%) lost to follow-up by 12 months (Group A: 3; Group B: 3). Table 1 (see PDF), descriptive statistics of preparation and restoration times, shows significantly longer preparation time in Group A (18.4 ± 3.2 min) than in Group B (12.6 ± 2.8 min; p < 0.001). Restoration times did not differ significantly (Group A: 9.2 ± 1.5 min; Group B: 8.8 ± 1.6 min; p = 0.12). Table 2 (see PDF) patient cooperation and anesthetic use highlights higher Frankl 4 ratings in Group A (85%) versus Group B (62%; p = 0.02). Anesthetic requirement was significantly lower in Group A (8%) compared to Group B (35%; p < 0.001). Table 3 (see PDF): 12-month USPHS evaluation demonstrates comparable retention (93.3% vs. 90.0%; p = 0.45), marginal adaptation (α ratings: 91.7% vs. 88.3%; p = 0.50) and absence of secondary caries (both 98.3%; p = 1.00).

## Discussion:

The present clinical trial demonstrates that Er: YAG laser cavity preparation, when combined with a self-adhesive flowable composite, achieves a clinical performance comparable to traditional rotary bur preparation and multistep adhesive protocols at the one-year follow-up. Notably, this combination also improved pediatric patient acceptance and reduced the requirement for local anesthesia, which is consistent with the laser's non-contact mode of action and reduced procedural discomfort [4]. The Er: YAG laser enables selective ablation of carious tissue while preserving healthy enamel and dentin. Its non-thermal, micro-explosive mechanism minimizes collateral damage and vibration, thus reducing procedural anxiety-a critical consideration in behavior management of pediatric patients [2, 6]. Previous studies have shown that laser-treated surfaces exhibit favorable morphological characteristics, such as micro-roughness and open dentinal tubules, which may enhance the mechanical interlocking of restorative materials despite potential concerns about insufficient demineralization or smear layer removal compared to acid etching [7]. Although the laser preparation duration exceeded that of conventional rotary instrumentation due to its lower ablation rate, this was effectively offset by the simplified restorative protocol of self-adhesive composites, which eliminates the need for separate etching, priming and bonding steps [3, 8]. This procedural streamlining is particularly beneficial in pediatric dentistry, where reducing chair-time and enhancing cooperation are paramount [9]. The study found no statistically significant differences between groups in terms of restoration retention, marginal integrity and color match, which align with existing literature suggesting that self-adhesive flowable composites demonstrate clinically acceptable performance in primary teeth at short- to medium-term follow-ups [10]. Despite the absence of phosphoric acid etching, the bond strength may be compensated by the laser-induced microretentive surface, which enhances micromechanical adhesion [11]. In terms of pediatric dental care, one of the key advantages of laser-assisted preparation is the reduction in local anesthetic usage, a factor that contributes to positive behavioral outcomes and facilitates better compliance during future appointments [12, 13]. This could be a decisive factor in treatment planning, especially for apprehensive children or those with a high dental anxiety index. However, the current study has limitations. Being a single-operator study, it may carry operator bias and the sample size was moderate, potentially limiting generalizability. Furthermore, the follow-up period of 12 months may not capture long-term restoration durability and secondary caries development. It is essential that future studies evaluate longer-term outcomes, involve multi-center cohorts and explore various laser energy settings and pulse durations to optimize ablation efficiency without compromising pulp vitality [14, 15]. Comparative trials with other bioactive or adhesive restorative systems, such as resin-modified glass ionomers or giomers, may provide broader insight into evidence-based minimally invasive dentistry [16, 17]. In summary, the combination of Er: YAG laser cavity preparation with self-adhesive flowable composites shows promise as a minimally invasive, child-friendly approach for treating occlusal caries in primary teeth, with clinical outcomes comparable to conventional methods and enhanced patient-centered benefits.

## Conclusion:

Er: YAG laser cavity preparation followed by self-adhesive flowable composite restoration offers an effective, child-friendly approach for treating carious primary molars, matching conventional methods in restoration longevity while improving patient cooperation and reducing anesthetic requirements.
